# Supplementary material for: Photoactive and Luminescent Transition Metal Complexes as Anticancer Agents: A Guiding Light in the Search for New and Improved Cancer Treatments
Source: Biomedicines. 2022 Mar 1;10(3):578. doi: 10.3390/biomedicines10030578 (PMC8945219; doi:10.3390/biomedicines10030578)
Supplement: Supplementary file 1 [file biomedicines-10-00578-s001.zip › biomedicines-1599302-supplementary.pdf]

biomedicines

MDPI

Supplementary Information

Luminescent transition metals as anticancer agents: A guiding light in the search for new and improved cancer treatments.

Brondwyn S. McGhie,[a] and Janice R. Aldrich-Wright[a*]

1 School of Science, , Nanoscale Organisation and Dynamics Group, Western Sydney University, Locked Bag 1797 Penrith South DC, 2751, NSW, Australia

* Correspondence: J.Aldrich-Wright@westernsydney.edu.au, Ph: +61246203218

Supplementary Table S1: Search results.

	Pt	Ru	Ir	Os	Rh	Other metals
Scopus	1 results as of 21/01/2022	0 results as of 21/01/2022	0 results as of 21/01/2022	0 results as of 18/01/22	0 results as of 18/01/22	
Web of science	15 results as of 21/01/2022	8 results as of 21/01/2022	14 results as of 21/01/2022	9 results as of 18/01/22	16 results as of 18/01/22	
PubMed	32 results as of 21/01/2022	28 results as of 21/01/2022	56 results as of 21/01/2022	2 results as of 18/01/22	9 results as of 18/01/22	
Total (after refining*)	21	23	33	8	14	14

* Removing duplicates and irrelevant papers.

Supplementary Table S2: Search strategy.

	Pt	Ru	Ir	Os	Rh
Scopus	(TITLE-ABS-KEY (Antineoplastic* OR Antineoplastics* OR Chemotherapeutic* OR (tumour AND Growth AND inhibition) OR Antitum* OR Chemotherapy* OR Anticancer* OR Cytotoxic* OR Cancer* OR anti*cancer OR chemotherap* OR anti*tum* OR anti*proliferat*)) AND (TITLE-ABS-KEY (Pt* OR Platinum* OR "Pt(II)"* OR "Pt(IV)"*)) AND (TITLE-ABS-KEY (photosensitive* OR light-sensitive* OR photoimageable* OR photo-imageable* OR photoresponsive* OR photo responsive* OR photo-responsive* OR light-sensitive* light*sensitive* OR photo*sensitive* OR lightsensitive* OR light*responsive* OR light*sensitive* OR	(TITLE-ABS-KEY (Chemotherapeutic* OR (tumour AND Growth AND inhibition) OR Antitum* OR Chemotherapy* OR Anticancer* OR Cytotoxic* OR Cancer*)) AND (TITLE-ABS-KEY ("Ru"* OR Ruthenium* OR "Ru(III)" * OR "Ru(II)" * OR "Ru(IV)" *)) AND (TITLE-ABS-KEY (photosensitive* OR light-sensitive* OR photoimageable* OR photo-imageable* OR photoresponsive* OR photo responsive* OR photo-responsive* OR light-sensitive* light*sensitive* OR photo*sensitive* OR lightsensitive* OR light*responsive* OR light*sensitive* OR photoelectric* OR photoconductor* OR photographic* OR light*sensing* OR	(TITLE-ABS-KEY (Chemotherapeutic* OR (tumour AND Growth AND inhibition) OR Antitum* OR Chemotherapy* OR Anticancer* OR Cytotoxic* OR Cancer*)) AND (TITLE-ABS-KEY ("Ir"* OR iridium* OR "Ir(III)" * OR "Ir(0)" * OR "Ir(IV)" *)) AND (TITLE-ABS-KEY (photosensitive* OR light-sensitive* OR photoimageable* OR photo-imageable* OR photoresponsive* OR photo responsive* OR photo-responsive* OR light-sensitive* light*sensitive* OR photo*sensitive* OR lightsensitive* OR light*responsive* OR light*sensitive* OR photoelectric* OR photoconductor* OR photographic* OR light*sensing* OR chemiluminescent* OR	(TITLE-ABS-KEY (Chemotherapeutic* OR (tumour AND Growth AND inhibition) OR Antitum* OR Chemotherapy* OR Anticancer* OR Cytotoxic* OR Cancer*)) AND (TITLE-ABS-KEY (Osmium* OR "Os(III)" * OR "Os(II)" * OR "Os(IV)" *)) AND (TITLE-ABS-KEY (photosensitive* OR light-sensitive* OR photoimageable* OR photo-imageable* OR photoresponsive* OR photo responsive* OR photo-responsive* OR light-sensitive* light*sensitive* OR photo*sensitive* OR lightsensitive* OR light*responsive* OR light*sensitive* OR photoelectric* OR photoconductor* OR photographic* OR light*sensing* OR chemiluminescent* OR	(TITLE-ABS-KEY (chemotherapeutic* OR (tumour AND growth AND inhibition) OR antitum* OR chemotherapy* OR anticancer* OR cytotoxic* OR cancer*)) AND (TITLE-ABS-KEY (rhenium* OR "Re(II)" * OR "Re(IV)" *)) AND (TITLE-ABS-KEY (photosensitive* OR light-sensitive* OR photoimageable* OR photo-imageable* OR photoresponsive* OR photo AND responsive* OR photo-responsive* OR light-sensitive* AND light*sensitive* OR photo*sensitive* OR lightsensitive* OR light*responsive* OR light*sensitive* OR photoelectric* OR photoconductor* OR

	photoelectric* OR photoconductor* OR photographic* OR light*sensing* OR chemiluminescent* OR phosphorescent* OR photoluminescent* OR fluorescent* OR luminescent*)) AND (TITLE-ABS-KEY ("CELL*LINE" OR "CELL*LINES" OR IC50 OR GI50 OR LD50 OR EC50 OR "in vitro"))	chemiluminescent* OR phosphorescent* OR photoluminescent* OR fluorescent* OR luminescent*)) AND (TITLE-ABS-KEY ("cell*line" OR "cell*lines" OR "in vitro"))	phosphorescent* OR photoluminescent* OR fluorescent* OR luminescent*)) AND (TITLE-ABS-KEY ("cell*line" OR "cell*lines" OR "in vitro"))	phosphorescent* OR photoluminescent* OR fluorescent* OR luminescent*)) AND (TITLE-ABS-KEY ("cell*line" OR "cell*lines" OR "in vitro"))	photographic* OR light*sensing* OR chemiluminescent* OR phosphorescent* OR photoluminescent* OR fluorescent* OR luminescent*)) AND (TITLE-ABS-KEY ("cell*line" OR "cell*lines" OR "in vitro"))
Web of science	(TI=(antineoplastic OR antineoplastic agents OR Chemotherapeutic OR Anticancer OR chemotherapy OR chemotherapy OR Chemotherapeutic OR Anticancer)) AND (TI=(photosensitive OR light-sensitive OR photoimageable OR photo responsive OR fluorescent OR photoelectric OR photoconductor OR photographic OR sensing OR chemiluminescent OR phosphorescent OR photoluminescent))AND (TI=(Platinum OR “Pt” or “Pt(II)” OR “Pt(IV)”))	(TI=(antineoplastic OR antineoplastic agents OR Chemotherapeutic OR Anticancer OR chemotherapy OR chemotherapy OR Chemotherapeutic OR Anticancer)) AND (TI=(photosensitive OR light-sensitive OR photoimageable OR photo responsive OR fluorescent OR photoelectric OR photoconductor OR photographic OR sensing OR chemiluminescent OR phosphorescent OR photoluminescent))AND (TI=(Ru OR Ruthenium OR "Ru(II)" OR "Ru(III)" OR "Ru(IV)"))	(TI=(antineoplastic OR antineoplastic agents OR Chemotherapeutic OR Anticancer OR chemotherapy OR chemotherapy OR Chemotherapeutic OR Anticancer)) AND (TI=(photosensitive OR light-sensitive OR photoimageable OR photo responsive OR fluorescent OR photoelectric OR photoconductor OR photographic OR sensing OR chemiluminescent OR phosphorescent OR photoluminescent))AND (TI=("Ir" OR iridium OR "Ir(III)" OR "Ir(0)" OR "Ir(IV)"))	((TS=(antineoplastic OR antineoplastic agents OR Chemotherapeutic OR Anticancer OR chemotherapy OR chemotherapy OR Chemotherapeutic OR Anticancer)) AND TS=(photosensitive OR light-sensitive OR photoimageable OR photo responsive OR fluorescent OR photoelectric OR photoconductor OR photographic OR sensing OR chemiluminescent OR phosphorescent OR photoluminescent)) AND TS= (Osmium OR "Os(II)" OR "Os(III)" OR "Os(I)")	((TS=(antineoplastic OR antineoplastic agents OR Chemotherapeutic OR Anticancer OR chemotherapy OR chemotherapy OR Chemotherapeutic OR Anticancer)) AND TS=(photosensitive OR light-sensitive OR photoimageable OR photo responsive OR fluorescent OR photoelectric OR photoconductor OR photographic OR sensing OR chemiluminescent OR phosphorescent OR photoluminescent)) AND TS=(Rhenium OR "Re(I)" OR "Re(IV)")
PubMed	(((photosensitive[All Fields] OR light-sensitive[All Fields] AND photoimageable[All Fields] or photo responsive[All Fields] AND photo[All Fields] OR photoelectric[All Fields] OR photoconductor[All Fields] OR photographic[All Fields] OR sensing[All Fields] AND ("semiconductors"[MeSH Terms] OR "semiconductors"[All Fields] OR "semiconductor"[All Fields]) OR chemiluminescent[All Fields] OR phosphorescent[All Fields] OR photoluminescen[All Fields]) AND (("antineoplastic agents"[Pharmacological Action] OR "antineoplastic agents"[MeSH Terms] OR ("antineoplastic"[All Fields] AND "agents"[All Fields]) OR "antineoplastic agents"[All Fields] OR "antineoplastic"[All Fields]) OR ("antineoplastic agents"[Pharmacological Action] OR "antineoplastic agents"[MeSH Terms] OR ("antineoplastic"[All Fields] OR "antineoplastic agents"[All Fields] OR "antineoplastics"[All Fields]) OR Chemotherapeutic[All Fields] OR	(((photosensitive[All Fields] OR light-sensitive[All Fields] AND photoimageable[All Fields] or photo responsive[All Fields] AND photo[All Fields] OR photoelectric[All Fields] OR photoconductor[All Fields] OR photographic[All Fields] OR sensing[All Fields] AND ("semiconductors"[MeSH Terms] OR "semiconductors"[All Fields] OR "semiconductor"[All Fields]) OR chemiluminescent[All Fields] OR phosphorescent[All Fields] OR photoluminescen[All Fields]) AND (("antineoplastic agents"[Pharmacological Action] OR "antineoplastic agents"[MeSH Terms] OR ("antineoplastic"[All Fields] AND "agents"[All Fields]) OR "antineoplastic agents"[All Fields] OR "antineoplastic"[All Fields]) OR ("antineoplastic agents"[Pharmacological Action] OR "antineoplastic agents"[MeSH Terms] OR ("antineoplastic"[All Fields] OR "antineoplastic agents"[All Fields] OR "antineoplastics"[All Fields]) OR Chemotherapeutic[All Fields] OR	(((photosensitive[All Fields] OR light-sensitive[All Fields] AND photoimageable[All Fields] or photo responsive[All Fields] AND photo[All Fields] OR photoelectric[All Fields] OR photoconductor[All Fields] OR photographic[All Fields] OR sensing[All Fields] AND ("semiconductors"[MeSH Terms] OR "semiconductors"[All Fields] OR "semiconductor"[All Fields]) OR chemiluminescent[All Fields] OR phosphorescent[All Fields] OR photoluminescen[All Fields]) AND (("antineoplastic agents"[Pharmacological Action] OR "antineoplastic agents"[MeSH Terms] OR ("antineoplastic"[All Fields] AND "agents"[All Fields]) OR "antineoplastic agents"[All Fields] OR "antineoplastic"[All Fields]) OR ("antineoplastic agents"[Pharmacological Action] OR "antineoplastic agents"[MeSH Terms] OR ("antineoplastic"[All Fields] OR "antineoplastic agents"[All Fields] OR "antineoplastics"[All Fields]) OR Chemotherapeutic[All Fields] OR	(((photosensitive[All Fields] OR light-sensitive[All Fields] AND photoimageable[All Fields] or photo responsive[All Fields] AND photo[All Fields] OR photoelectric[All Fields] OR photoconductor[All Fields] OR photographic[All Fields] OR sensing[All Fields] AND ("semiconductors"[MeSH Terms] OR "semiconductors"[All Fields] OR "semiconductor"[All Fields]) OR chemiluminescent[All Fields] OR phosphorescent[All Fields] OR photoluminescen[All Fields]) AND (("antineoplastic agents"[Pharmacological Action] OR "antineoplastic agents"[MeSH Terms] OR ("antineoplastic"[All Fields] AND "agents"[All Fields]) OR "antineoplastic agents"[All Fields] OR "antineoplastic"[All Fields]) OR ("antineoplastic agents"[Pharmacological Action] OR "antineoplastic agents"[MeSH Terms] OR ("antineoplastic"[All Fields] OR "antineoplastic agents"[All Fields] OR "antineoplastics"[All Fields]) OR Chemotherapeutic[All Fields] OR	(((photosensitive[All Fields] OR light-sensitive[All Fields] AND photoimageable[All Fields] or photo responsive[All Fields] AND photo[All Fields] OR photoelectric[All Fields] OR photoconductor[All Fields] OR photographic[All Fields] OR sensing[All Fields] AND ("semiconductors"[MeSH Terms] OR "semiconductors"[All Fields] OR "semiconductor"[All Fields]) OR chemiluminescent[All Fields] OR phosphorescent[All Fields] OR photoluminescen[All Fields]) AND (("antineoplastic agents"[Pharmacological Action] OR "antineoplastic agents"[MeSH Terms] OR ("antineoplastic"[All Fields] AND "agents"[All Fields]) OR "antineoplastic agents"[All Fields] OR "antineoplastic"[All Fields]) OR ("antineoplastic agents"[Pharmacological Action] OR "antineoplastic agents"[MeSH Terms] OR ("antineoplastic"[All Fields] OR "antineoplastic agents"[All Fields] OR "antineoplastics"[All Fields]) OR Chemotherapeutic[All Fields] OR

	Anticancer[All Fields] OR Anti-tumor[All Fields] AND Anti-tumour[All Fields] AND ("drug therapy"[Subheading] OR ("drug"[All Fields] OR "therapy"[All Fields]) OR "drug therapy"[All Fields] OR "chemotherapy"[All Fields]OR "chemotherapy"[All Fields]) OR Chemotherapeutic[All Fields] OR Anticancer[All Fields])) AND (("cell line"[MeSH Terms] OR "cell line"[All Fields]) AND ("cell line"[MeSH Terms] OR ("cell"[All Fields] AND "line"[All Fields]) OR "cell lineS"[All Fields] OR ("cell"[All Fields] AND "lines"[All Fields]) OR "cell lines"[All Fields]) OR GI50[All Fields] OR ("lethal dose 50"[MeSH Terms] OR "lethal dose 50"[All Fields] OR "ld50"[All Fields]) OR EC50[All Fields] OR ("in vitro techniques"[MeSH Terms] OR ("vitro"[All Fields] AND "techniques"[All Fields]) OR "in vitro techniques"[All Fields] OR "vitro"[All Fields] OR "in vitro"[All Fields])) AND (y_5[Filter])) AND (Platinum OR "Pt" or "Pt(II)" OR "Pt(IV)")	Anticancer[All Fields] OR Anti-tumor[All Fields] AND Anti-tumour[All Fields] AND ("drug therapy"[Subheading] OR ("drug"[All Fields] OR "therapy"[All Fields]) OR "drug therapy"[All Fields] OR "chemotherapy"[All Fields]OR "chemotherapy"[All Fields]) OR Chemotherapeutic[All Fields] OR Anticancer[All Fields])) AND (("cell line"[MeSH Terms] OR "cell line"[All Fields]) AND ("cell line"[MeSH Terms] OR ("cell"[All Fields] AND "line"[All Fields]) OR "cell lineS"[All Fields] OR ("cell"[All Fields] AND "lines"[All Fields]) OR "cell lines"[All Fields]) OR GI50[All Fields] OR ("lethal dose 50"[MeSH Terms] OR "lethal dose 50"[All Fields] OR "ld50"[All Fields]) OR EC50[All Fields] OR ("in vitro techniques"[MeSH Terms] OR ("vitro"[All Fields] AND "techniques"[All Fields]) OR "in vitro techniques"[All Fields] OR "vitro"[All Fields] OR "in vitro"[All Fields])) AND (y_5[Filter])) AND (Ru OR Ruthenium OR "Ru(II)" OR "Ru(III)" OR "Ru(IV)"))	Anticancer[All Fields] OR Anti-tumor[All Fields] AND Anti-tumour[All Fields] AND ("drug therapy"[Subheading] OR ("drug"[All Fields] OR "therapy"[All Fields]) OR "drug therapy"[All Fields] OR "chemotherapy"[All Fields]OR "chemotherapy"[All Fields]) OR Chemotherapeutic[All Fields] OR Anticancer[All Fields])) AND (("cell line"[MeSH Terms] OR "cell line"[All Fields]) AND ("cell line"[MeSH Terms] OR ("cell"[All Fields] AND "line"[All Fields]) OR "cell lineS"[All Fields] OR ("cell"[All Fields] AND "lines"[All Fields]) OR "cell lines"[All Fields]) OR GI50[All Fields] OR ("lethal dose 50"[MeSH Terms] OR "lethal dose 50"[All Fields] OR "ld50"[All Fields]) OR EC50[All Fields] OR ("in vitro techniques"[MeSH Terms] OR ("vitro"[All Fields] AND "techniques"[All Fields]) OR "in vitro techniques"[All Fields] OR "vitro"[All Fields] OR "in vitro"[All Fields])) AND (y_5[Filter])) AND ("Ir" OR iridium OR "Ir(III)" OR "Ir(0)" OR "Ir(IV)"))	Anticancer[All Fields] OR Anti-tumor[All Fields] AND Anti-tumour[All Fields] AND ("drug therapy"[Subheading] OR ("drug"[All Fields] OR "therapy"[All Fields]) OR "drug therapy"[All Fields] OR "chemotherapy"[All Fields]OR "chemotherapy"[All Fields]) OR Chemotherapeutic[All Fields] OR Anticancer[All Fields])) AND (("cell line"[MeSH Terms] OR "cell line"[All Fields]) AND ("cell line"[MeSH Terms] OR ("cell"[All Fields] AND "line"[All Fields]) OR "cell lineS"[All Fields] OR ("cell"[All Fields] AND "lines"[All Fields]) OR "cell lines"[All Fields]) OR GI50[All Fields] OR ("lethal dose 50"[MeSH Terms] OR "lethal dose 50"[All Fields] OR "ld50"[All Fields]) OR EC50[All Fields] OR ("in vitro techniques"[MeSH Terms] OR ("vitro"[All Fields] AND "techniques"[All Fields]) OR "in vitro techniques"[All Fields] OR "vitro"[All Fields] OR "in vitro"[All Fields])) AND (y_5[Filter])) AND (osmium OR "Os(II)" OR "Os(III)" OR "Os(IV)"))	Anticancer[All Fields] OR Anti-tumor[All Fields] AND Anti-tumour[All Fields] AND ("drug therapy"[Subheading] OR ("drug"[All Fields] OR "therapy"[All Fields]) OR "drug therapy"[All Fields] OR "chemotherapy"[All Fields]OR "chemotherapy"[All Fields]) OR Chemotherapeutic[All Fields] OR Anticancer[All Fields])) AND (("cell line"[MeSH Terms] OR "cell line"[All Fields]) AND ("cell line"[MeSH Terms] OR ("cell"[All Fields] AND "line"[All Fields]) OR "cell lineS"[All Fields] OR ("cell"[All Fields] AND "lines"[All Fields]) OR "cell lines"[All Fields]) OR GI50[All Fields] OR ("lethal dose 50"[MeSH Terms] OR "lethal dose 50"[All Fields] OR "ld50"[All Fields]) OR EC50[All Fields] OR ("in vitro techniques"[MeSH Terms] OR ("vitro"[All Fields] AND "techniques"[All Fields]) OR "in vitro techniques"[All Fields] OR "vitro"[All Fields] OR "in vitro"[All Fields])) AND (y_5[Filter])) AND (Rhenium OR "Re(II)" OR "Re(IV)"))

*Search terms "Os" and "Re" had to be removed from search terms as they elicited too many irrelevant papers for example "RE" to mean regarding and "OS" to stand for osteogenic sarcoma (cancer).
